# Supplementary material for: Shielding and activation of a viral membrane fusion protein
Source: Nat Commun. 2018 Jan 24;9:349. doi: 10.1038/s41467-017-02789-2 (PMC5783950; doi:10.1038/s41467-017-02789-2)
Supplement: Supplementary file 1 — Supplementary Information [file 41467_2017_2789_MOESM1_ESM.pdf]

Supplementary Table 1 | X-ray crystallography data acquisition and processing statistics.

	K_2PtCl_4 SAD data	Native data
**Data collection**		
Beamline	I03, Diamond Light Source	I03, Diamond Light Source
Resolution range (Å)	67.90–2.46 (2.52–2.46)*	48.99–1.60 (1.64–1.60)
Space group	$P2_12_12_1$	$P2_12_12_1$
Cell dimensions		
a, b, c (Å)	37.0, 94.4, 97.8	37.4, 94.2, 98.0
α, β, γ (°)	90.0, 90.0, 90.0	90.0, 90.0, 90.0
Wavelength (Å)	1.07146	0.97625
Unique reflections	10,034 (333)	46,607 (3,369)
Completeness (%)	76.7 (35.3)**	99.9 (99.6)
$CC_{1/2}$ (%)	99.9 (96.1)	99.8 (81.8)
R_{merge} (%)	16.7 (146.0)	8.6 (104.4)
I/σI	35.7 (2.6)	12.35 (2.0)
Avg. redundancy	129.5 (40.6)	9.7 (9.5)
**Refinement**		
Resolution range		48.99–1.60 (1.64–1.60)
Number of reflections		46,537 (4,565)
R_{work} (%)		16.5
R_{free} (%)***		19.9
**RMSD********		
Bonds (Å)		0.017
Angles (°)		1.52
**Model composition**		
Molecules per a.s.u		1
Atoms per a.s.u (protein/water)		2,331/336
**Average *B*-factors**		
Protein/water ($Å^2$)		34.3/40.5
**Ramachandran plot**		
Favoured (%)		99.0
Allowed (%)		1.0
Outliers (%)		0.0

* Numbers in parentheses refer to the relevant outer resolution shell.

** Completeness of data to 3.48 Å was 97.0 %

*** R_{free} equals the *R*factor as calculated above but using against 5% of the data removed prior to refinement.

**** RMSD: root mean square deviation from ideal geometry.

Supplementary Table 2 | Electron cryo microscopy data acquisition, processing and model fitting of fixed RVFV particles.

	RVFV	Hexamer 1	Hexamer 2	Hexamer 3	Pentamer
**Data acquisition**					
Frames per movie	88	N/A	N/A	N/A	N/A
Duration per frame (s)	0.2	N/A	N/A	N/A	N/A
Exposure rate (e^-/ pix / s)	2.5	N/A	N/A	N/A	N/A
Total exposure (e^- / Å^2)	22	N/A	N/A	N/A	N/A
Defocus* (µm)	1.0–3.0	N/A	N/A	N/A	N/A
**EMDB**	EMD-4197	EMD-4198	EMD-4199	EMD-4200	EMD-4201
**Data processing**					
Micrographs	943				
Particles**	2,995 (4,336)	55,710 (179,700)	28,445 (89,827)	24,009 (59,892)	23,762 (35,936)
Box size (pixels)	512	128	128	128	128
Symmetry	I1	C1	C2	C3	C5
Pixel size (Å)	2.7	2.7	2.7	2.7	2.7
Resolution (Å)***	13.3	8.0	8.6	8.0	7.7
B-factor applied	–800	–200	–200	–200	–200
**Fitting**					
Resolution (Å)	13.3	8.0	8.6	8.0	7.7
Model–map CC	0.96	0.87	0.90	0.87	0.88

* Positive value denotes underfocus.

** Number particles used for the reconstruction (numbers of all extracted particles are parenthesis).

*** Resolution determined by Fourier shell correlation at 0.143 threshold.

Supplementary Table 3 | Electron cryo-tomography data acquisition statistics.

	**RVFV pH 7.5**	**RVFV–liposome pH 7.5**	**RVFV–liposome pH 5.0**
Tilt range (°)	–30 – 60	–30 – 60	–30 – 60
Interval (°)	3	3	3
Frames per tilt	8	8	8
Duration per frame (s)	0.4	0.4	0.4
Exposure rate (e^-/ pix / s)	2.8	2.8	2.8
Total exposure (e^- / $Å^2$)	150	150	150
Defocus* (µm)	2.5–4.0	2.8–4.0	3.0–4.0
Tilt series	14	17	17
Viruses	120	103	30

* Positive value denotes underfocus.

Supplementary Table 4 | Electron cryo-tomography data processing and model fitting.

	Pentamer pH 7.5	Hexamer pH 7.5	Pentamer pH 7.5	Hexamer pH 7.5	Pentamer + memb. pH 7.5	Hexamer + memb. pH 7.5	Pentamer pH 5.0	Hexamer pH 5.0	Pentamer + memb. pH 5.0	Hexamer + memb. pH 5.0
**Sample**	RVFV	RVFV	RVFV–liposome	RVFV–liposome	RVFV–liposome	RVFV–liposome	RVFV–liposome	RVFV–liposome	RVFV–liposome	RVFV–liposome
**EMDB**	EMD-4202	EMD-4203	EMD-4204	EMD-4205	EMD-4206	EMD-4207	EMD-4208	EMD-4209	EMD-4210	EMD-4211
**Data processing**										
Sub-tomograms	1,417	13,011	903	9,502	348	1,716	259	2,300	94	904
Box size (pixels)	128	128	128	128	128	128	128	128	128	128
Pixel size (Å)	2.7	2.7	2.7	2.7	2.7	2.7	2.7	2.7	2.7	2.7
Symmetry	C5	C6	C5	C6	C5	C6	C5	C6	C5	C6
Resolution (Å)*	14	14	17	14	18	20	20	16	20	18
**Fitting**										
Resolution (Å)	20	N/A	N/A	N/A	N/A	N/A	20	N/A	20	N/A
Model–map CC	0.91	N/A	N/A	N/A	N/A	N/A	0.90	N/A	0.87	N/A

* Resolution determined by Fourier shell correlation at 0.143 threshold.

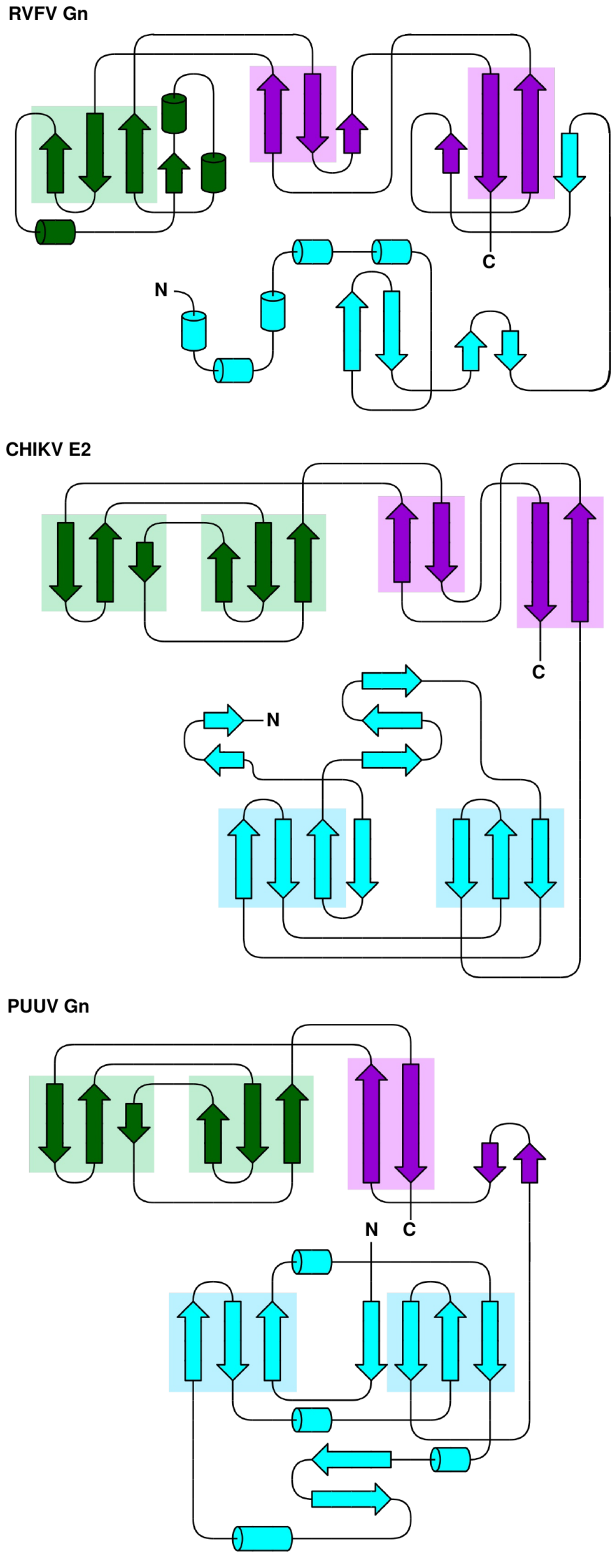

Supplementary Figure 1 | Secondary structural elements of fusion protein assembly partners. Topology diagrams show protein secondary structural elements of RVFV Gn, CHIKV E2 and PUUV Gn. Domains are coloured as in Figure 1 and termini are labelled C (carboxy-terminus) and N (amino-terminus). Shaded areas indicate elements which are shared between the different proteins. The CHIKV E2 and PUUV Gn share secondary structural elements in all three domains, sharing a total of 14 β-sheets. The RVFV Gn however has a unique domain A and secondary structural similarities are focused on domain B and the β-ribbon. The RVFV Gn and the CHIKV E2 share 7 β-sheets while the RVFV Gn and the PUUV Gn only share 5 β-sheets. The RVFV Gn is structurally more related to the CHIV E2 than the PUUV Gn based on secondary structural elements, but the CHIKV E2 and the PUUV Gn are structurally closer to each other than either are to the RVFV Gn.

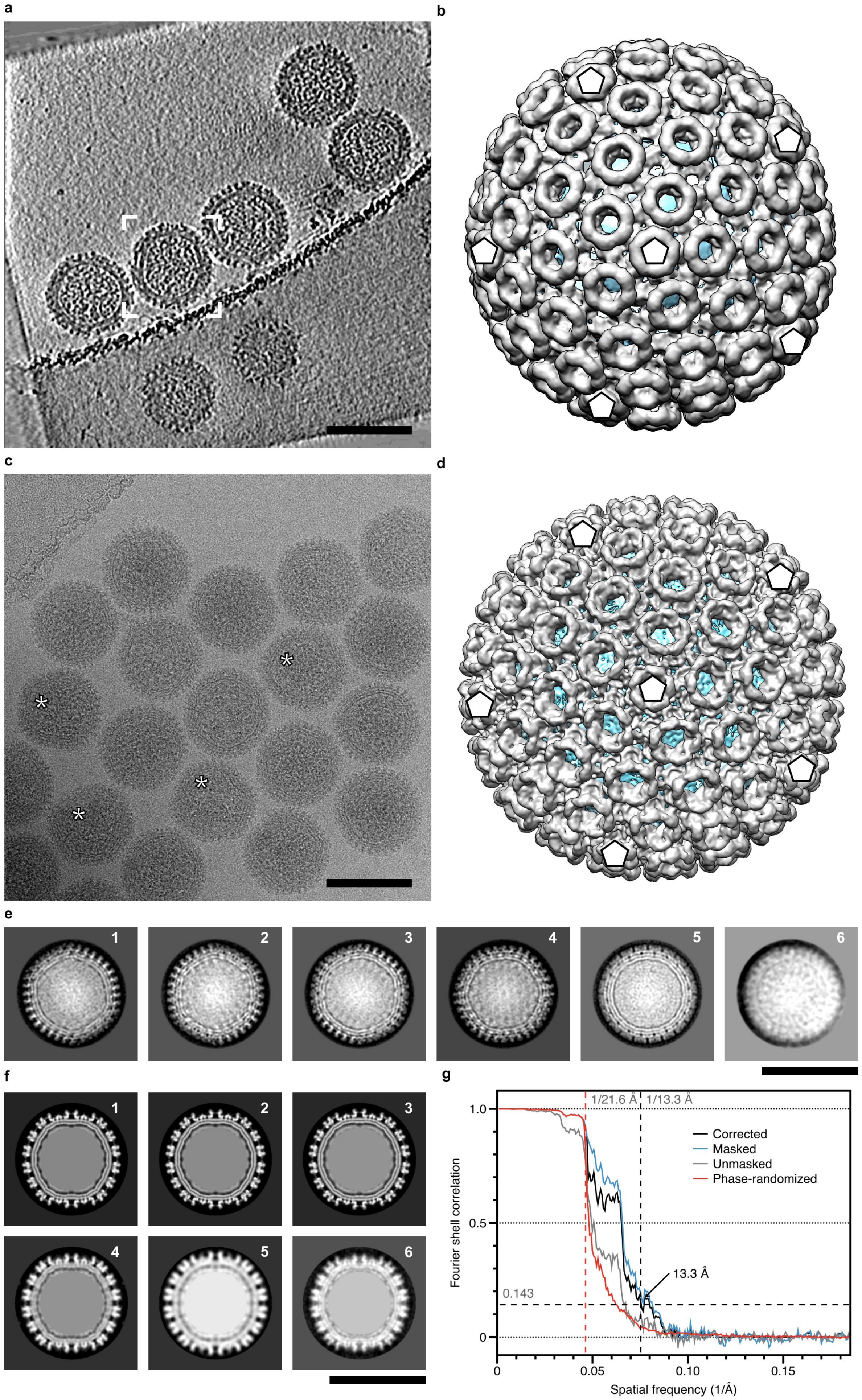
a
b
c
d
e
1
2
3
4
5
6
f
g
1/21.6 Å
1/13.3 Å
1.0
0.5
0
0.143
Fourier shell correlation
Corrected
Masked
Unmasked
Phase-randomized
13.3 Å
0
0.05
0.10
0.15
Spatial frequency (1/Å)

Supplementary Figure 2 | Electron cryomicroscopy of live and fixed RVFV particles. (**a**) An 8-nm thick slice through a tomogram showing several live RVFV particles. Particles are slightly oval and/or distorted in shape, suggesting they are flexible and deviate from perfect icosahedral symmetry. (**b**) The virus boxed in *a* was reconstructed by projecting capsomer sub-tomogram averages back onto their original locations on the virion surface. Pentamers on the reconstruction are indicated with white pentagons and the viral membrane is rendered light blue. Although the capsomers are organized on a *T*=12 icosahedral lattice in each virion, this arrangement may be flexible, reflected in the ellipsoidal shape of the displayed virion. (**c**) A micrograph of fixed RVFV particles. Fixation helps to retain the icosahedral shape of the particles. Particles that deviate from icosahedral symmetry (asterisks) are still present and such particles were excluded from further analysis. (**d**) Single particle reconstruciton of fixed RVFV particles at 13.3-Å resolution is shown along the five-fold axis of icosahedral symmetry. The view is roughly the same as in *b* and pentamers are indicated with white pentamers. The arangement of the pentamers and hexamers is consistent with *T*=12 icosahedral lattice. (**e**) Representative two-dimensional class averages of RVFV particles showing the flexible nature of the particles. Class-averages were showed two-fold (1–3), three-fold (4) and five-fold (5) views, consistent with icosahedral symmetry. (**f**) Cross sections of 3D class averages (6 representatives from the total of 10 are shown; one class was empty). Particles contributing to classes 1–3 were included in the final reconstruction. The exterior of the particle and the interior density corresponding to the viral genome have been masked out. Scale bars, 100 nm. (**g**) Fourier shell correlation was calculated without (gray) and within (blue) a mask defining the glycoproteins shell of the particle and plotted to Nyquist frequency (1/5.4 Å). The contribution of the mask to correlation was taken into account by phase-randomization (red) to yield a corrected curve (black) using Relion (*relion_postprocess*). The spatial frequency at which the correlation drops below the thershold (0.143) is marked with a black dashed line. Estimated resolution of 13.3 Å is indicated.

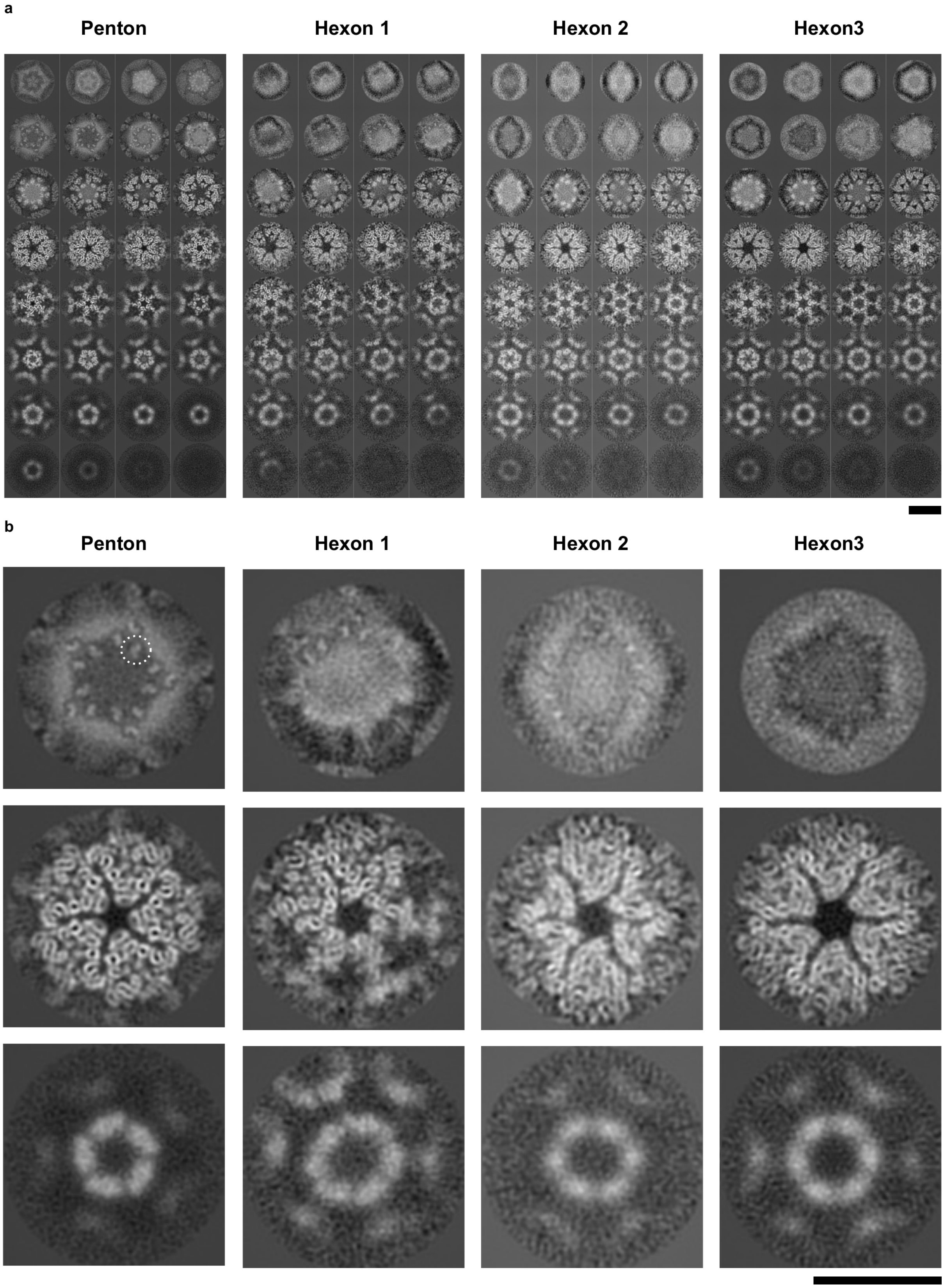

Supplementary Figure 3 | Localized reconstructions of the four different capsomer types. (**a**) Density is shown as slices (2.7 Å thick) for each of the four capsomer types (penton, hexon type 1, 2 and 3). Every second slize is shown from the virion interior (top left corner) to the virion surface (lower right corner). (**b**) Four-times magnified close-ups of selected slices from the space between the two leaflets of the lipid bilayer (top row), from the floor of the glycoprotein shell (middle row) and the tips of the capsomers (bottom row). One example are with transmembrane densities is circled with a dashed line. Scale bars, 20 nm.

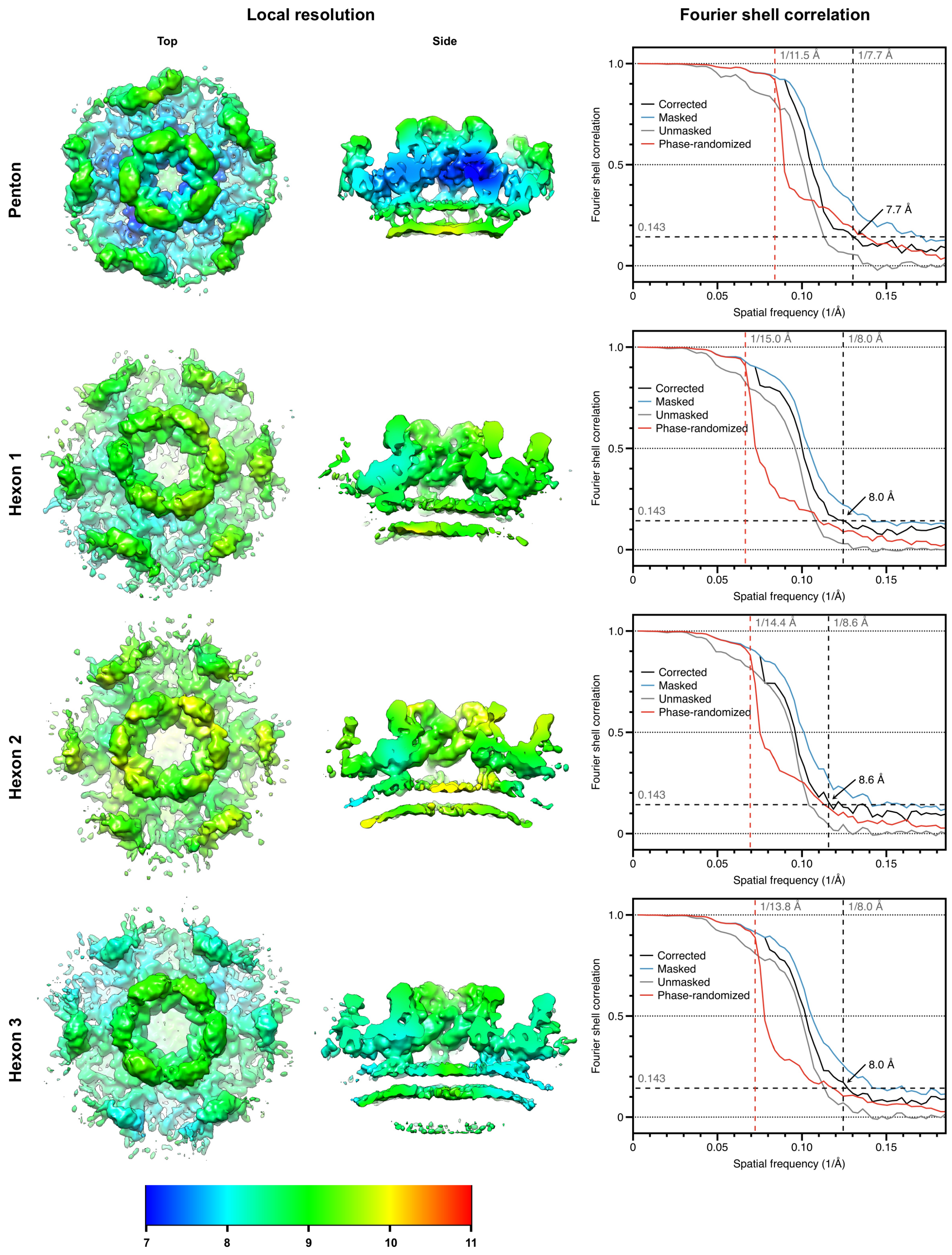

Local resolution
Top
Side
Penton
Hexon 1
Hexon 2
Hexon 3
7
8
9
10
11
Fourier shell correlation
Fourier shell correlation
Spatial frequency (1/Å)
Corrected
Masked
Unmasked
Phase-randomized
1/11.5 Å
1/7.7 Å
7.7 Å
1/15.0 Å
1/8.0 Å
8.0 Å
1/14.4 Å
1/8.6 Å
8.6 Å
1/13.8 Å
1/8.0 Å
8.0 Å
0.143

Supplementary Figure 4 | Resolution of the localized reconstructions. (**a**) Density for each capsomer type (penton, hexon type 1, 2 and 3) is shown from the top (left column) and from the side (right column). In the side-view only a slab of density is shown for clarity. The surface of each reconstruction is coloured according to local resolution as estimated by Resmap software. The local resolution of membrane-proximal regions of the glycoprotein layer is generally better than membrane distal regions. The pentamer is the best resolved capsomer with regions showing local resolution of 7 Å. (**b**) Resolution of the four different capsomer types estimated by Fourier shell correlation is plotted. For each resolution assesment the reconstruction was masked with a soft mask (width of mask edge 3 pixels) generated in Relion (*relion_postprocess*). Due to the fact that the density unavoidably extends outside of the mask edge in the localized reconstrutions, the masking operation introduced overestimation of resolution (blue curves). This overestimation was compensated for using the purpose-built phase-randomization test in Relion (*relion_postprocess*; red curves). The spatial frequency at which phase-randomization was started is indicated with a dashed red line. After this frequency the area above zero is due to mask contribution. Subtraction of the mask contribution allowed deriving corrected resolution estimates for the capsomers (black curves). The spatial frequency at which the corrected correlation drops below the threshold (0.143) is indicated with a black dashed line and the corresponding resolution estimate is indicated.

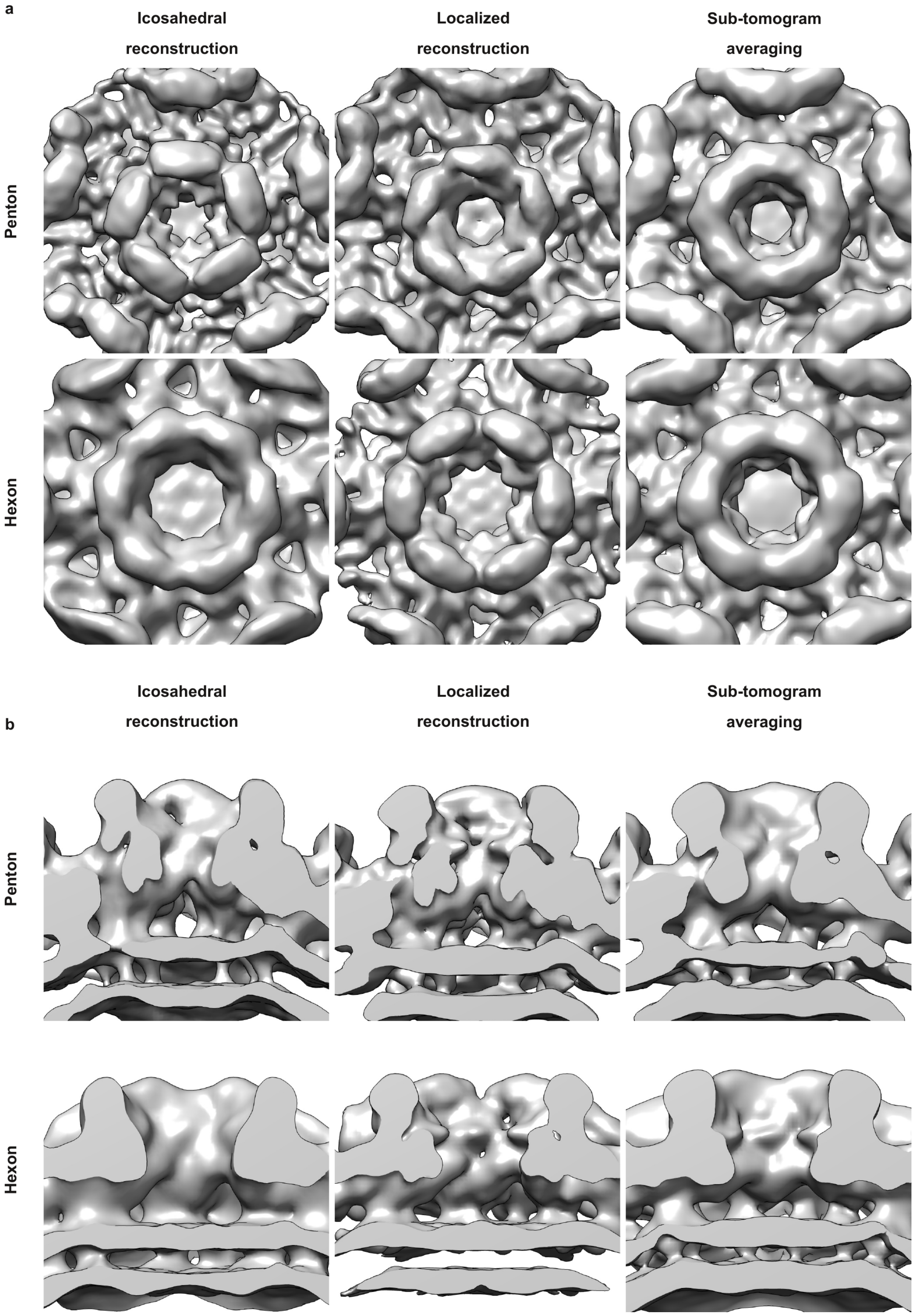

Supplementary Figure 5 | Comparison of reconstructions from different methods. (**a–b**) Views of the pentameric and hexameric capsomers from the top (*a*) and from the side (*b*) are shown to compare the different reconstruction methods. Type 2 hexon was used in the comparison in the case of icosahedral and localized reconstruction. Maps from icosahedral and localized reconstructions have been lowpass filtered to 14-Å resolution to allow direct comparison to the sub-tomogram averages that were resolved to this resolution.

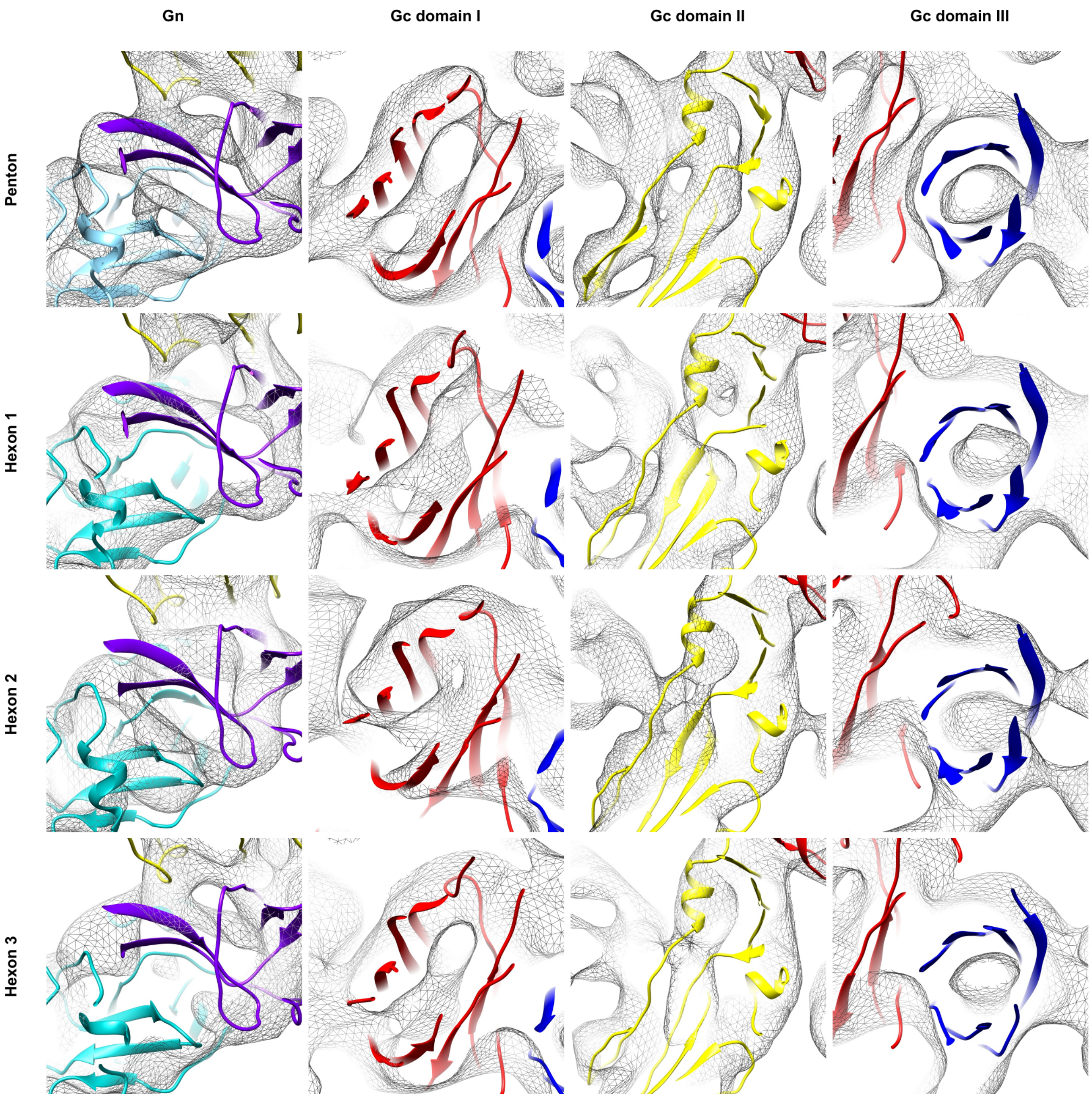

Supplementary Figure 6 | Flexible fitting of the Gn and Gc crystal structures into cryo-EM densities from localized reconstruction. Crystal structures of RVFV Gn and Gc were fitted into densities of the four different capsomers solved by localized reconstruction. The fitted atomic models are shown in ribbon and coloured as in Figure 2. The cryo-EM surface are shown as a mesh. The fittings of Gn, and Gc domains I–III are shown for the penton and each three types of hexons. The Gn and Gc are mostly β-type proteins and fittings reveal an excellent fit of β-sheets in their corresponding cryo-EM densities**.**

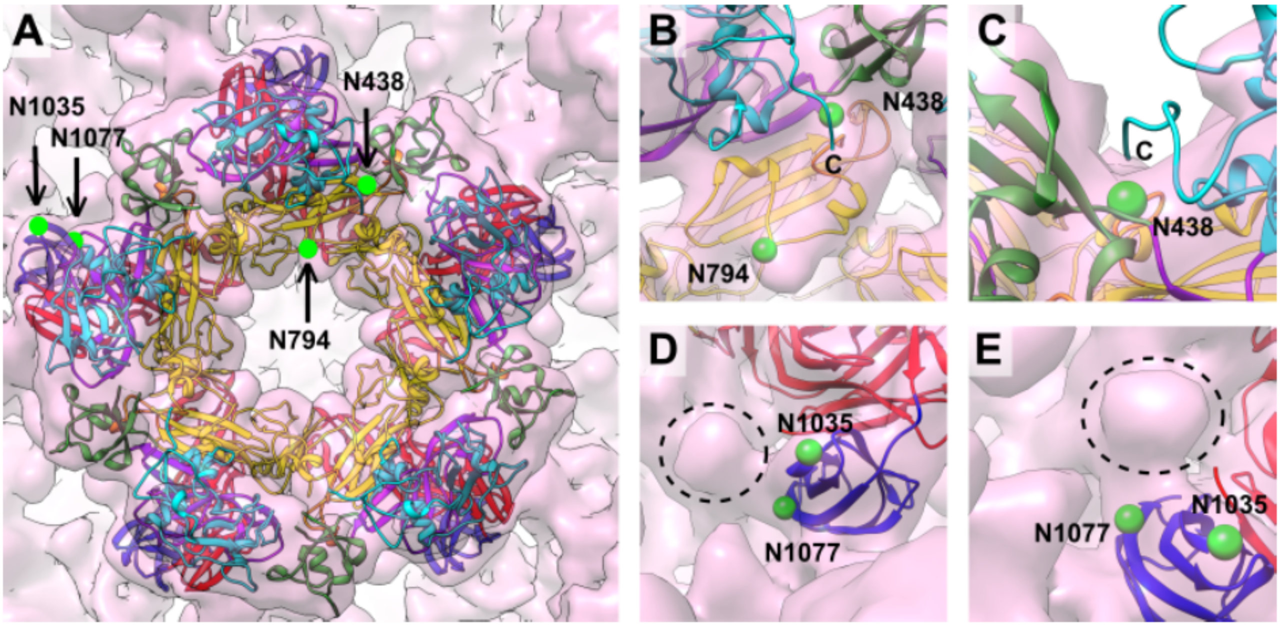

Supplementary Figure 7 | Positions of the putative N-linked glycan of Gn and Gc. (A) An overview of the pentamer model fitted in the localized reconstruction (pink surface). Glycosylation sites are labeled and represented by green spheres. (**B**) The N438 (of the Gn) and N794 (of the Gc) glycosylation sites are surface exposed and face into the central cavity of the capsomers. A putative glycan at N438 of Gn would be close to the C-terminus of Gn. (**C**) The density corresponding to the C-terminus of Gn is not resolved which may indicate that it is flexible. (**D–E**) Putative Gc glycans N1035 and N1077 are found in the inter-capsomer region. N1035 is solvent exposed while N1077 close to a globular density (indicated with a dashed line), which may be occupied by this glycan.

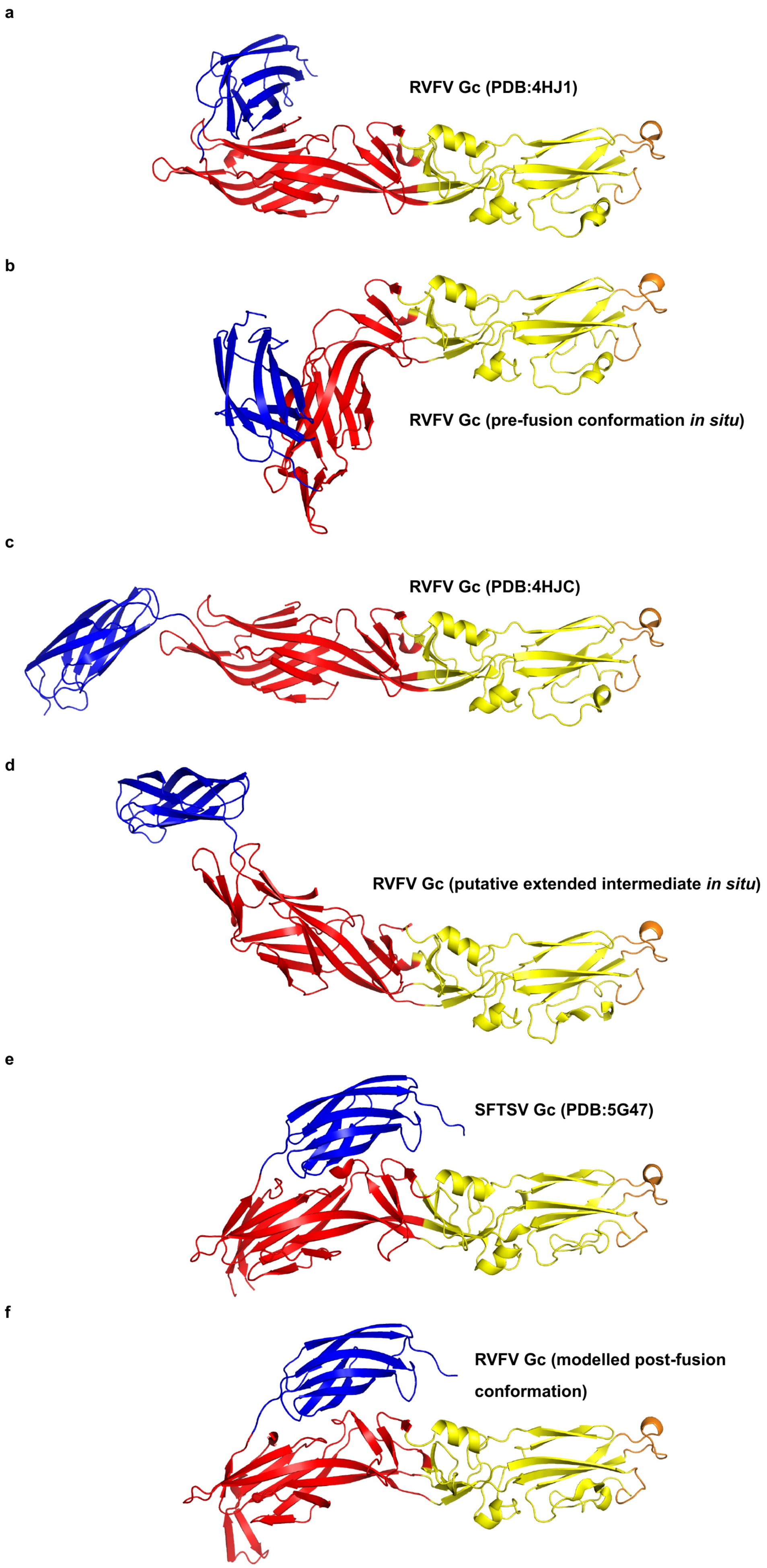
a
RVFV Gc (PDB:4HJ1)
b
RVFV Gc (pre-fusion conformation *in situ*)
c
RVFV Gc (PDB:4HJC)
d
RVFV Gc (putative extended intermediate *in situ*)
e
SFTSV Gc (PDB:5G47)
f
RVFV Gc (modelled post-fusion conformation)

Supplementary Figure 8 | Different conformations of RVFV Gc. (**a**) The structure of Gc as captured by X-ray crystallography (PDB:4HJ1). (**b**) A pre-fusion conformation as observed on the virion surface by single particle cryo-EM and MDFF fitting at pH 7.5. (**c**) A putative extended intermediate state observed by X-ray crystallography (PDB:4HJC). (**d**) An extended intermediate observed on the virion surface by tomography and MDFF at pH 5.0 in the presence of a target membrane. (**e**) A post-fusion state from a closely related phlebovirus, sever fever with thrombocytopenia syndrome virus (SFTSV, 25% sequence identity, PDB:5G47) captured by X-ray crystallography. (**f**) A post-fusion homology model of the RVFV Gc based on the SFTSV post-fusion structure, created with SWISS-MODEL software. All models are shown in ribbon and coloured according to domain as in Figure 2. PDB accession numbers are given for published models.

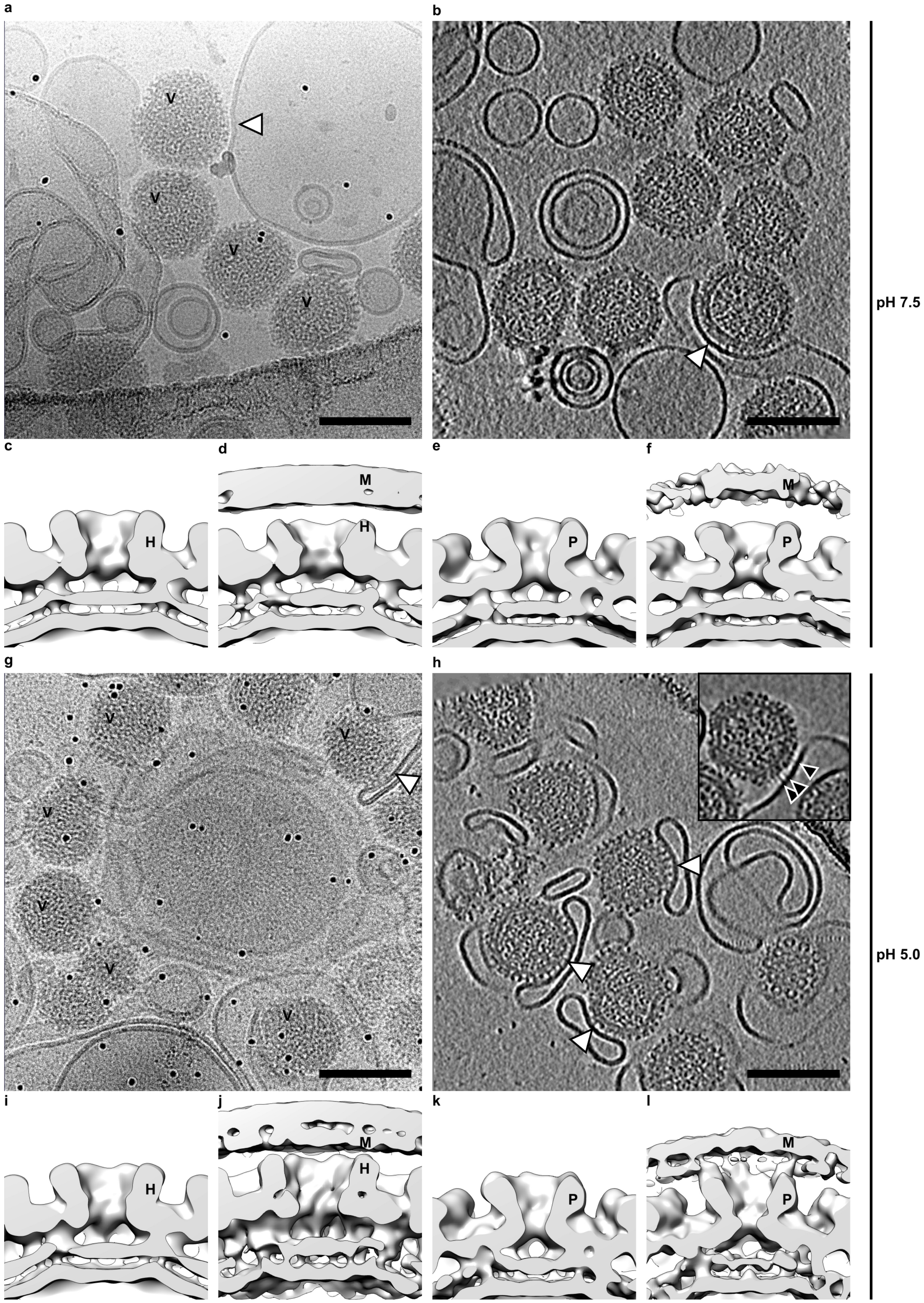
a
b
V
V
V
V
c
d
e
f
M
M
H
H
P
P
pH 7.5
g
h
V
V
V
V
V
V
i
j
k
l
M
M
H
H
P
P
pH 5.0

Supplementary Figure 9 | Electron cryomicroscopy and tomography of RVFV–liposome mixtures. (**a**–**b**) Two-dimensional projection image (*a*) and 8-nm thick tomographic density section (*b*) are shown for RVFV–liposome mixtures at pH 7.5. Scale bars, 100 nm. (**c**–**f**) Sub-tomogram averages of hexameric (H) and pentameric (P) capsomers from samples at pH 7.5 with and without a target membrane (M). (**g**–**h**) Two-dimensional projection image (*g*) and 8-nm thick tomographic density section (*h*) are shown for RVFV–liposome mixtures at pH 5.0. The inset in *h* shows bridge-like densities (black arrowheads) connecting the virus and liposome membrane. Scale bars, 100 nm. (**i–l)**, Sub-tomogram averages of hexameric (H) and pentameric (P) capsomers from samples at pH 5.0 with and without a target membrane (M). Some RVFV virions (V) are labelled in *a* and *g*. RVFV–liposome contacts zones are labelled in *a*, *b*, *g* and *h* with white arrowheads. Whilst the contact zones occur both at pH-neutral control and in the acidified samples, subtomogram averages reveal the membrane insertion of the pentameric capsomers only in the acidified sample (*j*). All sub-tomogram averages have been low-pass filtered to 20-Å resolution for comparison.

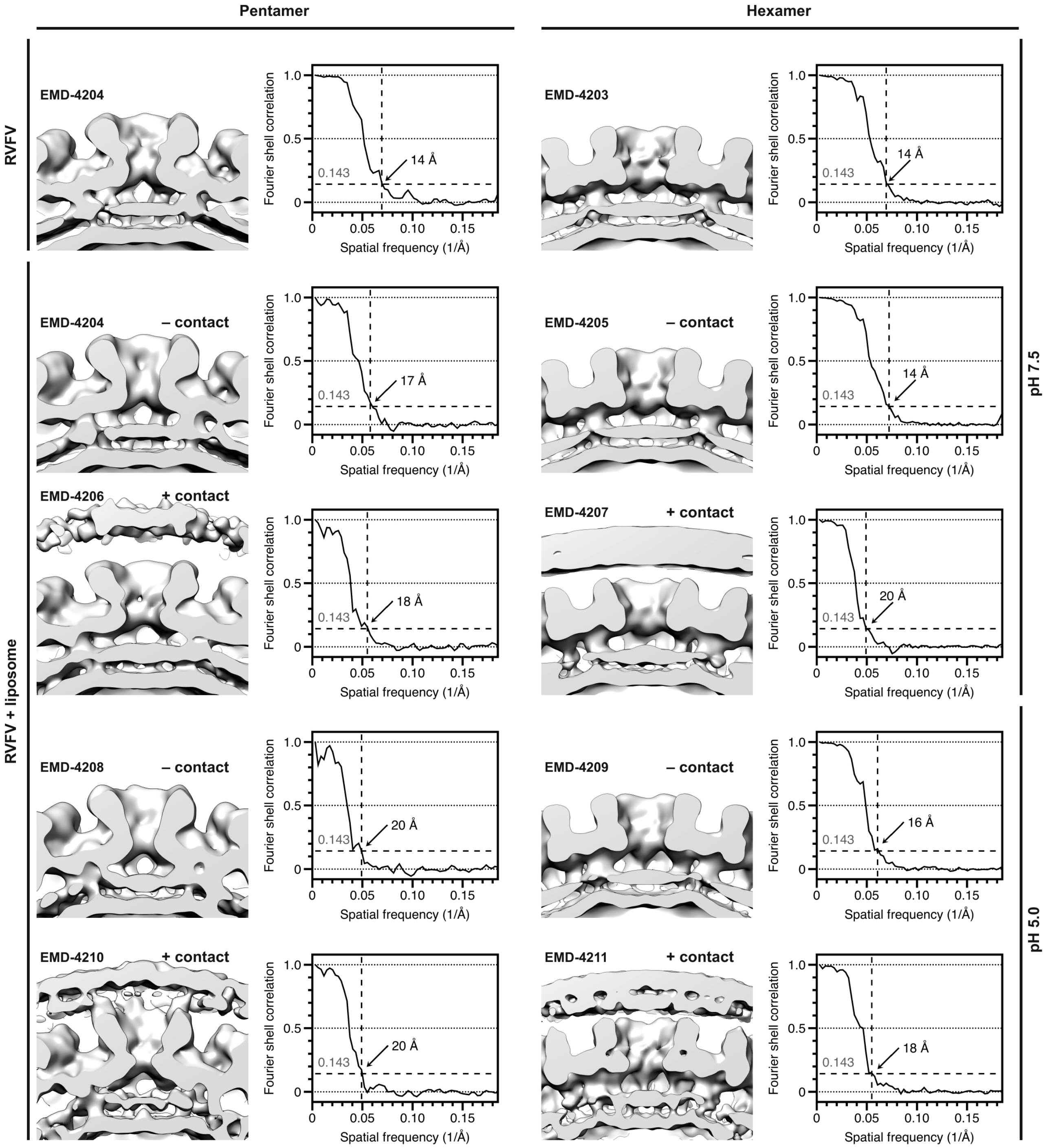

Supplementary Figure 10 | Resolution estimation of sub-tomogram averages from purified RVFV and RVFV–liposome mixtures. Fourier shell correlation (FSC) is plotted for each of the ten sub-tomogram averages. Pentameric capsomers are shown on the left and hexameric capsomers on the right. The resolution corresponding to the spatial frequency at which the FSC drops below the theshold (0.143) is indicated in each plot. The type of the sample used (RVFV virions alone [RVFV] or mixtures of virions and liposomes [RVFV+liposomes]) is indicated on the left of the figure. The pH of the sample is indicated on the right or the figure. In the RVFV+liposome samples, the presence (+ contact) and absence (– contact) of the target membrane in the averaged structure is indicated. All of the structures are independent from one another and their comparison provides also a useful cross-validation. The EMDB codes are indicated for each of the averages.

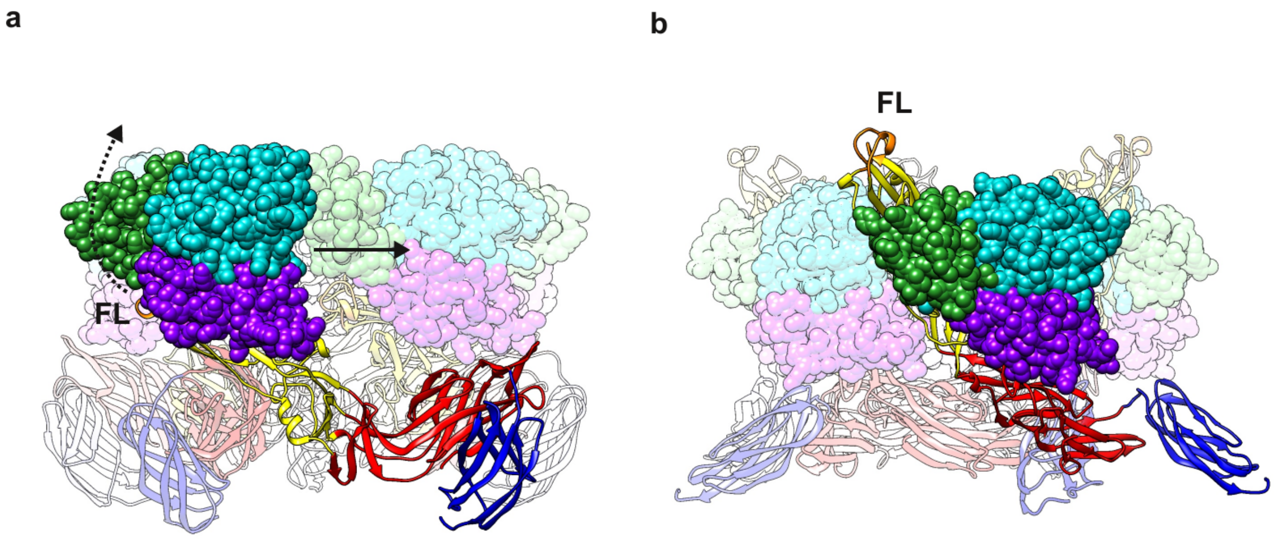

Supplementary Figure 11 | Model for unshielding of the fusion loops by exposure to acidic pH in the presence of a target membrane, derived by subtomogram averaging and fitting of atomic models. (**a**) Model of the pentamer in the prefusion state. Unshielding of the fusion loops (FL) is achieved at acidic pH by movement of the Gn (space fill model; solid arrow). This leads to extension of the Gc (ribbon) and exposure of the FL (dashed line). (**b**) Model of the membrane facing pentamer at acidic pH.

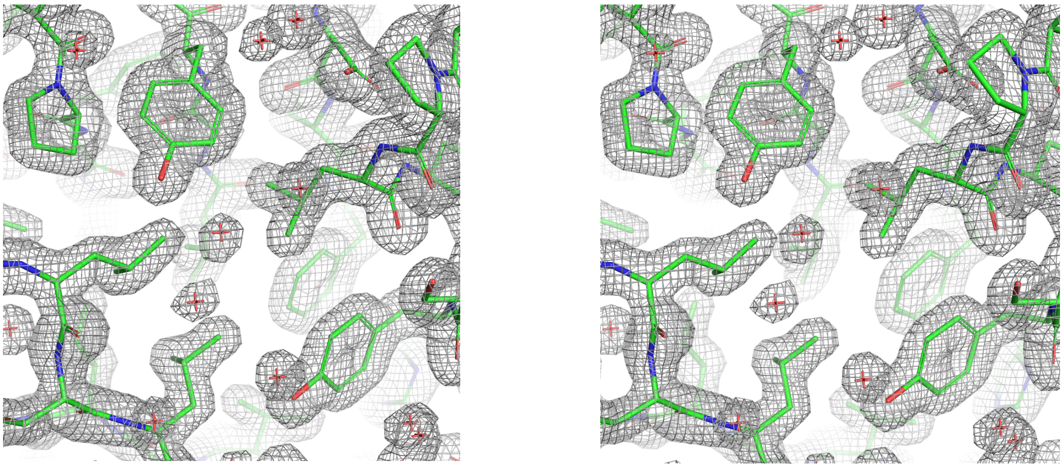

Supplementary Figure 12 | Stereo image for a portion of the Gn electron density. The density is shown as a mesh contoured from a 2Fo–Fc map at 1 sigma.
