# Supplementary material for: Shielding and activation of a viral membrane fusion protein
Source: Nat Commun. 2018 Jan 24;9:349. doi: 10.1038/s41467-017-02789-2 (PMC5783950; doi:10.1038/s41467-017-02789-2)
Supplement: Supplementary file 3 — Description of Additional Supplementary Files [file 41467_2017_2789_MOESM3_ESM.pdf]

File Name: Supplementary Movie 1

Description: Quasi-atomic model of Rift Valley fever virus. A quasi-atomic model of the virion surface was created by calculating a localized reconstruction of the pentameric capsomer and using flexible fitting to fit the crystallographic structures of glycoproteins Gn and Gc in the density. The same fitting was repeated for the localized reconstructions of the hexameric capsomers. Icosahedral symmetry was applied on the chains creating the asymmetric unit of the virion to create a full atomic model of the glycoprotein surface consisting of 720 Gn–Gc heterodimers.
